# Romanian ophthalmologists in the renowned ophthalmic journals worldwide

**Published:** 2018

**Authors:** Dan Călugăru, Mihai Călugăru

**Affiliations:** * Department of Ophthalmology, University of Medicine and Pharmacy, Cluj-Napoca, Romania; ** OcuCenter Ophthalmological Clinic, Cluj-Napoca, Romania

**Keywords:** central/hemicentral retinal vein occlusion, malignant glaucoma, diabetic macular edema, neovascular age-related macular degeneration

## Abstract

The authors are discussing over 80 articles that they have published in the last 5 years in the renowned ophthalmic journals worldwide, which have approached, for the first time in the ophthalmic literature, the following 4 topics: the acute central/ hemicentral retinal vein occlusion, the therapeutic interventions in the fellow eye of patients with unilateral malignant glaucoma, the persistent diabetic macular edema, and the current researches in patients with neovascular age-related macular degeneration.

***The aim of this presentation is to detail the researches we have carried out during the period 2013-2019. The passages relevant to the topics discussed in this article were taken from the international journals where we had published these researches, indicating strictly the source of acquisition (authors, title of the journal, year, volume, issue, pages)***.

In the last 5 years we have published over 80 ISI articles (Institute of Scientific Information; Thompson Reuters Publishing House) visible on the Web of Science, including the following 4 topics, which we have approached for the first time in the ophthalmic literature: the acute central/ hemicentral retinal vein occlusion (central/ hemicentral RVO), the therapeutic interventions in the fellow eye in patients with unilateral malignant glaucoma, the pathogenic factors in the diabetic macular edema (DME), and the current researches in patients with neovascular age-related macular degeneration (nAMG). 

## 1. The acute central/ hemicentral RVO

***1.1 Definition and diagnostic criteria of acute ischemic central/ hemicentral RVO***

The term “acute” occlusion was suggested by Hayreh et al. [**1**], who subdivided the venous occlusions into the following three stages according to the length of time between onset and examination of the eye: the early acute stage of the disease when the eye was examined within 90 days, the intermediate stage when it was examined 91-365 days after the occlusion onset, and the late stage when the examination of the eye was performed more than 1 year since the onset of venous occlusion. Unfortunately, most of the current studies are conducted in patients with intermediate and late stages of venous occlusions. All the patients included in our researches had acute central/ hemicentral RVOs whose duration of symptoms was ≤ 1 month after the occlusion was diagnosed. 

The diagnostic criteria for the ischemic type of acute central/ hemicentral RVOs were determined based on the angiography result. In cases with angiographically clear evidence of retinal capillary nonperfusion zones, the criteria included 10 or more disc areas of nonperfusion. If intraretinal hemorrhages prevented a clear angiographic evaluation of retinal capillary nonperfusion, we considered the following parameters: a best-corrected visual acuity (BCVA) score ≤ 20/ 400 Snellen equivalent; ability to see ≤ V/ 4e isopter based on the Goldmann perimeter; the presence of relative afferent pupillary defects in patients with a normal fellow eye; extensive ocular fundus changes (striking amount of hemorrhages, venous tortuosity, cotton wool spots [>5], disc and macular edema); and an intraocular pressure (IOP) reduction in the occluded eye of ≥ 4 mmHg compared with the congener eye. An eye was classified as having ischemic central/ hemicentral RVO by the presence of at least 4 of these 5 parameters [**2**-**4**].

***1.2 Treatment of acute central/hemicentral RVOs***

Initially, the treatment for acute central/ hemicentral RVO patients consisted of 4 consecutive intravitreal bevacizumab (Avastin; Genentech Inc., San Francisco, CA) injections (IVB) administered off-label at a dose of 2.5 mg per injection, with each injection spaced approximately 45 days apart. Thereafter, IVB therapy was flexible, and subsequent injections were administered during the scheduled visits whenever a visual acuity loss of ≥ 5 early treatment diabetic retinopathy study (ETDRS) letters occurred and/ or iris/ angle neovascularization (NV) appeared, regardless of the IOP level. Panretinal photocoagulation (PRP) was performed as soon as intraocular NV was diagnosed, unless it subsided after 2 consecutive IVB injections, administered 30 days apart, and topical steroids and cycloplegics were prescribed. In cases of elevated IOP, topical fixed combination of timolol and dorzolamide (FCTD; Cosopt, Merck & CO., Inc., Whitehouse Station, NJ) was added. Unless IOP normalized in response with these treatments, surgery was advised, after administering an additional IVB injection [**2**,**5**].

***1.3 Three-year outcomes of a prospective clinical study***

Our prospective clinical study on the 3-year results of bevacizumab treatment at a dose of 2.5 mg (0.1 ml) in patients with acute (≤ 1 month after the occlusion was diagnosed) central/ hemicentral RVOs substantiated for the first time evidence suggesting that early treatment applied immediately after the clinical onset of venous occlusion provided significant and sustained improvements in BCVA and central macular thickness (CMT) with inactive disease (dry retina and stable visual acuity [VA] for at least 6 months after the last injection) in most phakic patients with acute central/ hemicentral RVO, making this treatment option a rational and viable therapeutic strategy. Specifically, the BCVA improved with a mean of 17.15 and 26.81 ETDRS letters in nonischemic and ischemic occlusions, respectively, and sustained retina dryness was achieved in 91.23% of the patients. There were 2 mild cases of NVG, which rapidly reversed after treatment, and 5 cases with macular edema caused by subretinal fluid, that resolved after IVB injections, with rapid restoration of macular morphology [**2**,**6**-**20**]. We documented that the burden of frequent intravitreal injections could be significantly reduced and the longer intervals with improved macular edema could be significantly provided by an increase of the dose of bevacizumab to 2.5 mg (0,1 ml). The total number of injections of bevacizumab administered on a period of 36 months was 9.14. There were no adverse effects or ocular toxicity, including clinically evident sterile or infectious endophthalmitis, IOP increase, retinal ruptures, retinal detachment, and systemic thromboembolic events, during the study [**21**]. Bevacizumab was more effective in patients with ischemic occlusions, who required a significantly higher number of injections than the nonischemic forms (a mean of 9.7 and 8.7 injections, respectively). To our knowledge, the assessment of the visual results of IVB treatment in patients with acute central/ hemicentral RVOs, who were followed for at least 3 years, had not been previously reported. 

***1.4 Greater visual gains after treatment in patients with poor baseline VA***

We documented, for the first time, in patients with macular edema secondary to acute central/ hemicentral RVOs treated with bevacizumab [**2**], that patients with poor baseline VA generally experienced greater visual gains after treatment compared with their counterparts with better vision. Specifically, on month 36, the proportions of VA increases (from baseline values) were 36% in nonischemic occlusions with better initial VA and 352.7% in ischemic occlusions having a poor initial VA. We attributed this finding to the fact that patients with better VA at the time of treatment typically had a treatment “ceiling effect” as a consequence of the limited potential for improvement.

***1.5 Is narrow angle a risk factor for acute central/ hemicentral RVO?***

In 2014, we prospectively evaluated, for the first time, the gonioscopic findings, and their changes during a 3-year follow-up period in 57 patients with acute central/ hemicentral RVO [**42**]. Our results showed that 21% of the patients with central/ hemicentral RVOs presented with narrow angles and the rest had normal angles. Ocular globes with narrow angles had a mean axial length and anterior chamber depth that were significantly smaller and a mean cornea thickness that was significantly greater compared with eyes with normal angles. In eyes with narrow angles, the retinal vein and artery, which share the same adventitial sheath, are more crowded and impaired as they pass through lamina cribrosa. This status may narrow the lumen of the vein, with all its subsequent consequences, namely, decreased blood flow, increased blood viscosity, and local turbulence that could cause thrombosis. That is why, a narrow angle in the context of a significantly smaller ocular globe than that of the normal average eye may represent a local risk factor predisposing a patient to central/ hemicentral RVO, especially for the ischemic form of venous occlusion. Intermittent episodes of angle closure may contribute to the occurrence of central/ hemicentral RVO as well as to the progression of the gonioscopic findings from primary angle closure suspect (PAC suspect) to primary angle closure (PAC) and from PAC to primary angle closure glaucoma (PACG) [**22**]. 

***1.6 Prevention of neovascular glaucoma (NVG)***

We prospectively evaluated, for the first time, the cumulative prevalence of NVG in 57 patients with acute (≤ 1 month after the occlusion was diagnosed) central/ hemicentral RVOs treated with IVB injections [**2**]. In 2 cases of ischemic central retinal vein occlusion, NVG was diagnosed in the 12th and 18th months of the follow-up period. These subjects presented with an open anterior chamber angle, iris NV, and IOPs of ≤ 45 mmHg; they rapidly reversed after the IVB injections, associated with topical steroids, cycloplegics, and FCTD. Because IOP normalized and iris NV disappeared after treatment, the only valid criterion we used for reinjection was a visual loss of ≥ 5 ETDRS letters. Accordingly, administration of IVB injections continued until stabilization of the BCVA score lasting ≥ 6 months was achieved. Therefore, the first case of NVG received 10 IVB injections and the second, 9 injections with no serious ocular or systemic adverse events. Because NV had disappeared, PRP had not been administered. The IOP was maintained within statistically normal values, with topical FCTD. So, the rate of the cumulative prevalence of NVG was 4.08% [**23**,**24**]. Additionally, a comparison group was structured after the post-hoc analysis of an observational study published in 1987 [**25**]. At the end of the follow-up, the cumulative prevalence of NVG was 28.2%, a value significantly different from that existing in our patients with treated occlusions (4.08%). We believe that IVB at a dose of 2.5 mg injected promptly before occurrence of neovascularization and IOP elevation offers a real benefit and promise for the prevention of NVG in patients with acute central/ hemicentral RVOs. 

***1.7 Acute central/ hemicentral RVO, ocular hypertension (OH), and central corneal thickness (CCT)***

Most of the patients in our series [**26**] with OH and associated central/ hemicentral RVOs presented a thicker CCT, with the average value being significantly greater (565.46 ± 13 μm) (P = 0.002) than that in the patients without OH (546.09 ± 30.23 μm). Because a thin CCT represents an independent, well-known risk factor for conversion of OH to POAG, we considered that a thicker CCT could have an inverse effect e.g., a protective effect against the development of glaucoma. The following could explain the protective effect of a thicker CCT: higher IOP values than those existing in reality, as measured by applanation tonometry, that require a more aggressive ocular hypotensive therapy; more rigid optic nerve architecture (including lamina cribrosa), that is less likely to develop a glaucomatous lesion; and less distensibility and elasticity of the ocular tissues [**26**].

***1.8 Conclusion***

We considered acute central/ hemicentral RVO an ophthalmic emergency. Therefore, immediate and aggressive therapy with anti-vascular endothelial growth factor (VEGF) agents is essential and imperative. The sooner the treatment is initiated, the sooner the patient is likely to have improvement in VA and foveal thickness. Every delay in treatment will adversely affect restoration of visual functions, which are difficult to restore even with subsequent treatment. Regardless of the intravitreal pharmacotherapy chosen, namely, specific (bevacizumab/ ranibizumab [Lucentis; Genentech Inc.]/ aflibercept [Eylea, Regeneron Pharmaceuticals, Tarrytown, NY, USA]) or nonspecific (dexamethasone implant) anti-VEGF agents, the treatment paradigms used (e.g., treat-and-extend/ pro re nata/ fixed-interval/ escalated algorithm), the patient age, and the form of central/ hemicentral RVO (ischemic/ nonischemic occlusion), the efficacy of treatment depends primarily on the promptness of the therapy after the onset of venous occlusion. Both groups of anti-VEGF substances provide similar rates of vision improvement using the current algorithms for administration, but with superior anatomic outcomes and fewer injections in the dexamethasone implant-treated eyes. However, more patients receiving the dexamethasone implant lose vision mainly due to cataract [**27**-**41**].

## 2. The therapeutic interventions in the fellow eye of patients with unilateral classical malignant glaucoma (MG)

We divided for the first time the fellow eyes of patients with unilateral classical MG into three groups:

***2.1 Eyes of patients who meet the diagnostic criteria for PAC suspect*** [**42**,**43**]

These eyes are apparently normal e.g., the angle is completely open but narrow with normal visual functions and IOP. However, these eyes have a great risk to develop an acute or subacute attack of angle closure in the future, given the biometric similarity with the other eye that has already experienced a MG. The optimal intervention for prophylaxis of MG is peripheral iridotomy/ iridectomy, which should be carried out promptly, immediately after the start of the appropriate therapy for unilateral classical MG. If the surgery is performed at the stage of apparently normal eye with entirely open angle and normal IOP, MG does not occur postoperatively in spite of the disease in the other eye [**43**,**44**].

***2.2 Eyes of patients who fulfill the diagnostic criteria of PAC*** [**42**,**43**] 

If some of the angles are already closed and the IOP is increased, the most intensive medical treatment should be carried out in an attempt to open the angle and to lower the tension in preparation for iridectomy [**43**,**44**]. MG occurs only in eyes in which some or all of the angles are closed preoperatively. Surgical intervention has to be performed in these eyes without a malignant postoperative reaction, if preoperatively the angle is open or has been opened entirely by intensive medical therapy. The tension at the time of surgery is an unreliable guide to the likelihood of MG occurrence. We recommend a peripheral iridotomy/ iridectomy or trabeculectomy depending on the level of the IOP reached after medical treatment, namely, the IOP normalized or it remained elevated, respectively [**43**,**44**].

***2.3 Eyes of patients who meet the diagnostic criteria of PAC glaucoma*** [**42**,**43**] 

In most cases, the primary chronic irreversible angle-closure glaucoma of the fellow eye occurs in eyes predisposed to angle-closure by their small dimensions with shorter axial length, shallower anterior chamber, thicker sclera, and a relatively larger lens. We documented, for the first time [**44**], the possibility of evolution of the primary chronic irreversible long-standing angle-closure glaucoma toward a malignant pre-glaucoma and even to a primary MG. The mechanisms involved in this process include expansion of choroidal volume by an accumulation of serous fluid in the extravascular choroidal space, slackness of lens zonules, and poor conductivity of fluid through the vitreous [**45**] owing to prolonged angle-closure as well as to severe long-standing intraocular inflammation. All these factors cause the vitreous and lens to move forward creating a ciliovitreolenticular block with posterior pooling of aqueous in the vitreous or behind it. In these cases [**44**], we recommend a combined operation e.g., pars-plana aspiration (with removal of liquid or liquefied vitreous), trabeculectomy and phacoemulsification-intraocular lens implantation if the lens is opaque. If pars-plana aspiration fails to extract liquid from the vitreous cavity, pars-plana vitrectomy is mandatory.

***2.4 Conclusion***

The fellow eye of the patients with unilateral classical MG is markedly predisposed to develop the MG after surgery. It can be managed successfully by appropriate and timely interventions.

## 3. The persistent DME

***3.1 Pathogenic factors***

The patients suffering from persistent DME have previously been treated with anti-VEGF and/ or corticosteroid intraocular injections, grid and scatter laser photocoagulation with insufficient macular deturgescence. Most likely, there was a permanent VEGF receptor 2 – mediated breakdown of the inner blood-barrier and a permanent VEGF receptor 1 – mediated rupture of the retinal pigment epithelium junctions induced by long-term VEGF overexpression and high vitreous level of placental growth factor in patients with DME. This permanent chronic retinal capillaropathy (pigmentary changes in the fovea, poorly controlled severe recurrent macular edema, telangiectatic vessels with leakage, and epiretinal membrane formation) caused by long-standing duration of DME and diabetes was temporarily relieved by reduction of the edematous component with antiangiogenic treatment. However, the pathology was incurable owing to irreversible ischemic changes to the macular ganglion cell complex, close to the foveola, with macular edema being a minor factor. VEGF is one proven contributor to macular edema in patients with diabetic retinopathy. Besides, a lot of proinflammatory and proangiogenic cytokines, chemokines, and growth factors may be associated with pathophysiology of DME, suggesting that the pathogenesis of DME is not only related to VEGF dependency. The whole panoply of these pro-permeability factors could be included in this latter group of possible contributors to DME in addition of VEGF, which were maximally expressed in the ischemic lesions of the long-standing DME and which exacerbated the deterioration primarily caused by VEGF in the initially damaged macular ganglion cell complex [**46**-**50**].

***3.2 Treatment***

We believe that the specific anti-VEGF drugs (e.g., bevacizumab/ ranibizumab/ aflibercept) represent the front-line therapy for the treatment of DME, but VEGF inhibition alone may be not sufficient to suppress the inflammatory response. Therefore, addition of a non-specific anti-VEGF substance, e.g., intravitreal steroid injection, which inhibits the upregulation of VEGF and suppresses the expression of the whole inflammatory factors is mandatory. Otherwise, patients will be impeded to achieve maximal visual and anatomic benefits [**51**-**58**]. 

***3.3 Conclusion***

The efficacy of therapy depends primarily on the precociousness of the treatment after DME diagnosis. Therefore, therapy with antiangiogenic agents has to be promptly applied as soon as possible after DME onset. Every delay of therapy adversely influences the deterioration of visual functions, which are difficult to restore, even with subsequent treatment [**59**-**66**].

## 4. The current researches in patients with nAMD 

***4.1 Potential adverse effects of anti-VEGF therapy***

Anti-VEGF therapy might be one of the potential determinants of the macular atrophy (MA) because it can interfere with the maintenance of the ocular vasculature of the normal retina and choriocapillaris. In the treatment of nAMD with antiangiogenic agents, two adverse effects of aflibercept should be considered and accounted for. Specifically, unlike bevacizumab, which has a protective effect against occlusion of choriocapillaris induced by photodynamic therapy [**67**], and ranibizumab, which does not impair the choroidal thickness [**68**], aflibercept treatment may result in a significant subfoveal choroidal thickness loss [**68**], by suppressing the choroidal vascular hyperpermeability and vasoconstriction, as well as by more pronounced reductions of choriocapillaris endothelium thickness and number of fenestrations. The thinning of the choroid consisted of the loss of small and medium vessels with baring of larger vessels, as well as the loss of pigmented cells, with clumping of preserved pigmented cells in various regions of the choroid. On short-term, the significant subfoveal choroidal thickness thinning by aflibercept does not seem to result in visual deleterious changes. However, on long-term, the prolonged inhibition of VEGF using aflibercept may affect the integrity of the choriocapillaris, considering the key role of VEGF-A in the normal function of the retina and in the regulation of the survival and permeability of the choriocapillaris. Thus, choroidal vascular impairment may affect the integrity of the retinal pigment epithelium (RPE) and outer retina favoring the development of the fovea-involving geographic atrophy (GA) with subsequent visual damaging effects because the choroid is involved in maintaining the perfusion of the outer retinal layers and is the sole source of metabolic exchange (nourishment and oxygen) for the fovea. Of note, excessive drying of the retina after treatment with anti-VEGF agents may promote the development of the GA. The presence of subretinal fluid is associated with a lower incidence of GA, and the presence of sub-RPE fluid is associated with slower growth of GA [**69**]. In addition, through the fragment crystallizable (Fc) domain, aflibercept can bind to the Fc receptor of both choriocapillaris endothelial cells and red blood cells, leading to complement-mediated cell death [**70**-**76**].

***4.2 Development of GA in patients with treated nAMD***

Because VEGF plays an important role in the normal function of the retina and the maintenance of the choriocapillaris by the RPE, therapies that block VEGF could have an effect of the development and progression of GA. Pathogenesis of the MA in treated nAMD (located foveal/ extrafoveal, within the bed of previous choroidal NV, in close proximity or clearly outside the area of total choroidal NV lesions) is currently unclear and may or may not be distinct from GA that develops in the setting of de novo GA lesions (purely dry AMD). It is not known whether their histology, growth patterns, and functional effects are similar to those of de novo GA lesions that develop in areas where no NV has been present previously. Atrophic lesions associated with treated NV are clinically indistinguishable from the GA that most clinicians historically think of as arising in dry AMD [**77**,**78**]. 

***4.3 Conclusion***

Regardless of the intravitreal pharmacotherapy chosen, namely specific (e.g. bevacizumab/ ranibizumab/ aflibercept) or nonspecific (e.g. corticosteroid implant) anti-VEGF agents, the treatment dosing paradigms chosen (e.g. treat-and-extend/ pro re nata/ fixed-interval/ escalated algorithm), the patient age, the baseline visual acuity, and the angiographic type, the efficacy of the treatment depends primarily on the promptness of the therapy after the nAMD diagnosis (onset). Therefore, therapy with antiangiogenic agents has to be promptly applied as soon as possible after nAMD onset. Every delay of therapy adversely influences the deterioration of visual function, which is difficult to restore even with subsequent treatment. The rationale for the use of dexamethasone implant in neovascular AMD includes decrease in VEGF-production and release, depletion in leukocytes migration, downregulation of several proinflammatory cytokines, prostaglandins, and intercellular adhesion molecule-1 expression, and restoring the integrity of the blood-retinal barrier (antipermeability effect) [**79**-**89**]. 

**Financial interest**

None of the authors has any financial interest in the subject matter of this paper.

**Acknowledgement**

The authors would like to thank Prof. Mircea Filip, MD, PhD, President of the Romanian Society of Ophthalmology, for the inclusion of the Romanian Society of Ophthalmology in the One Network of the American Academy of Ophthalmology, which has facilitated our access to the most prestigious ophthalmology journals in the world.

**Disclosures**

None.
